# Pattern and determinants of contraceptive use among the muslim women in Wajir and Lamu counties in Kenya: a cross-sectional study

**DOI:** 10.1186/s12905-024-02892-9

**Published:** 2024-01-18

**Authors:** Batula Abdi, Jerry Okal, Gamal Serour, Vincent Were, Marleen Temmerman, Peter Gichangi

**Affiliations:** 1 United Nations Population Fund, Uganda Country Office, Kampala, Uganda; 2 Department of Public Health and Primary Care, Faculty of Medicine and Health Sciences, Ghent University, Ghent, Belgium; 3 Population Council, Nairobi, Kenya; 4 International Islamic Center for Population Studies and Research, Al Azhar University, Cairo, Egypt; 5 Data Synergy and Evaluation unit, African Population and Health Research Center Nairobi, Nairobi, Kenya; 6 Department of Public Health and Primary Care, Faculty of Medicine and Health Sciences, Ghent University, Ghent, Belgium; 7 Centre of Excellence Women and Child Health, Aga Khan University, Nairobi, Kenya; 8 Technical University of Mombasa, Mombasa, Kenya; 9 Department of Public Health and Primary Care, Faculty of Medicine and Health Sciences, Ghent University, Ghent, Belgium

**Keywords:** Family Planning, Contraceptives, Muslim communities

## Abstract

**Background:**

Improving access to family planning (FP) is associated with positive health benefits that includes averting nearly a third of all maternal deaths and 10% of childhood deaths. Kenya has made great strides in improving access to family planning services. However, amid this considerable progress, regional variation has been noted which begs the need for a clearer understanding of the the patterns and determinants that drive these inconsistencies.

**Methods:**

We conducted a cross-sectional study that involved 663 Muslim women of reproductive age (15–49 years) from Wajir and Lamu counties in Kenya between March and October 2018.The objective of this study was to understand patterns and determinants of contraceptive use in two predominantly Muslim settings of Lamu and Wajir counties that have varying contraceptive uptake. Eligible women were interviewed using a semi-structured questionnaire containing socio-demographic information and history of family planning use. Simple and multiple logistic regression were used to identify determinants of family planning use. The results were presented as Crude Odds Ratio (COR) and Adjusted Odds Ratio (AOR) ratios at 95% confidence interval. A *p*-value of 0.05 was considered statistically significant.

**Results:**

Of the 663 Muslim women of reproductive age consenting to participate in the study, 51.5%, *n* = 342 and 48.5%, *n* = 321 were from Lamu and Wajir County, respectively. The prevalence of women currently using contraceptive was 18.6% (*n* = 123). In Lamu, the prevalence was 32.8%, while in Wajir, it was 3.4%. The determinants of current contraceptive use in Lamu include; marital status, age at marriage, employment status, discussion with a partner on FP, acceptability of FP in culture, and willingness to obtain information on FP. While in Wajir, determinants of current contraceptive use were education, and the belief that family planning is allowed in Islam.

**Conclusions:**

Our study found moderately high use of contraceptives among Muslim women of reproductive age in Lamu county and very low contraceptive use among women in Wajir. Given the role of men in decision making, it is critical to design male involvement strategy particularly in Wajir where the male influence is very prominent. It is critical for the government to invest in women and girls’ education to enhance their ability to make informed decisions; particularly in Wajir where FP uptake is low with low education attainment. Further, our findings highlight the need for culturally appropriate messages and involvement of religious leaders to demystify the myths and misconception around family planning and Islam particularly in Wajir.

**Supplementary Information:**

The online version contains supplementary material available at 10.1186/s12905-024-02892-9.

## Introduction

Globally there has been clear commitment to improve access to quality family planning services. The 2030 agenda for sustainable development has two relevant targets to family planning under the broader goals of health and wellbeing (SDG 3) and on gender equality and the empowerment of women and girls ( SDG 5) [1]. Despite impressive progress globally, contraceptive use in Africa is worryingly low at 36% [1, 2] compared to the Caribbean, North America, and Latin America that have a prevalence rate of over 70% [1].

The public health importance of family planning is well documented. Improving access to family planning in countries with high fertility rates can potentially reduce poverty and hunger and avert 32% of all maternal deaths and nearly 10% of childhood deaths [3]. Family planning has benefits that go beyond the health sector. Ensuring couples and individuals have a choice to decide how many and how frequent they will have children will considerably contribute to women’s empowerment, the achievement of universal primary schooling, and long-term environmental sustainability [4].

Kenya has made great strides in improving access to family planning services. In 2021, the government of Kenya renewed its commitment to ensure all citizens have equitable access to affordable quality family planning. The government aims to ensure there is availability of FP services to the last mile, increase modern contraceptive prevalence rate for married women from 58 to 64%, reduce unmet family planning need to 10% and transform social norms and eliminate social cultural barriers related to FP access by 2030 [5].

The number of women using contraceptives has steadily increased over time. In the past decade, the Contraceptive Prevalence Rate (CPR) increased from 32% in 2003 to 39% in 2009, 53% in 2014 and 58% in 2023 [6, 7]. Despite the increase in CPR, there has been progressive reduction in unmet need for family planning from 28% in 1998 to 26% in 2009, 18% in 2014 and 14% in 2023 [6, 7]. In spite of this progress, there are huge regional variations in CPR. The CPR in some of the regions in Kenya (including the central region with a CPR of 76%) is comparable to that of developed countries while some regions (such as North Eastern) have recorded CPR as low as 3.4% [7]. Wajir County has a CPR of 3%, which is one of the lowest in the country. On the other hand, Wajir county has the highest TFR in Kenya of eight children per woman [5] while Lamu County has a CPR of 39% and a TFR of 4 children per woman [6].

Studies have examined factors that determine the use of FP. The predominant factors include age, education, place of residence; social-economic status; spousal communication, method related concerns, access related issues including health provider’s attitudes and religion [7–10]. Several studies have established that FP utilization is highest among women who are educated and those aged 25–35 [11–13]. It has also been cited that girls education has a knock-on effect on age at first marriage, entrance into the paying labour market, and correlates with fertility reduction [14–16]. Similarly, women who are economically empowered or who are employed or have a source of income are more likely to use FP [10, 17]. Additionally, evidence shows that spousal communication has a positive impact on FP use [18–20].

Several studies have been conducted to establish whether contraceptive use differed with religious beliefs. While this has been the centre of debate with regard to FP use, a qualitative study conducted in Lamu and Wajir County shows that there is a widespread misinterpretation of Islam with regards to the permissibility of contraceptive use [21]. Studies have shown that Islam permits family planning. It is evident from the various fatwas^1^ (declarations) in the Muslim countries and evidence from various sources of Islamic sharia^2^ (law) that family planning is permitted in Islam [22, 23]. Despite the evidence on the permissibility of contraception in Islam, there is still a misconception among many Muslim communities that Islam does not permit contraception [21, 22, 24].

Apart from the national surveys, such as demographic health surveys, there is a paucity of data on determinants of family planning uptake among the Muslim community in Kenya. Furthermore, the Kenya Demographic Health Survey (KDHS) does not provide a detailed analysis of why the uptake of FP is so low among Muslims. The objective of this study is to understand patterns of contraceptive use and determinants of contraceptive use in the two predominantly Muslim settings of Lamu and Wajir counties that have varying contraceptive uptake. Studies have shown that uptake of family planning could be influenced by religious beliefs and cultural practices. Understanding the determinants of contraceptive use in these settings is important for informing the design of culturally appropriate family planning programmes.

## Methods

### Study design and setting

This was an analytical cross-sectional household survey conducted between March and October 2018 in Wajir and Lamu counties. Two sub-counties were purposefully selected in each county, namely Wajir East and Wajir North in Wajir County, and Lamu West and Lamu East in Lamu County. The process of selecting the sub-counties was informed by consultations with the district health management team, with careful attention given to factors such as the balance between rural and urban areas and security consideration. During the period of data collection, both counties were confronted with security concerns, thus the District health management teams recommended the two sub counties in each county that were considered safe.

### Study setting

Wajir county is ranked as one of the poorest in the country with as many as 76% of its residents having low education levels and only 4% have completed secondary level education or higher [25]. In Lamu, nearly a third of the county’s residents live below the poverty line with only 13% having competed secondary education [25]. The primary school gross enrolment rates for Wajir and Lamu is 55% and 80% respectively whereas the secondary gross enrolment rates for the two counties are 22% and 36% respectively [26]. Lamu County is a predominantly coastal community of the Swahili ethnic group while Wajir County is inhabited primarily by pastoralist Somalis.

The two counties are mainly inhabited by Muslim communities and have distinct sources of origin for the spread of Islam. In Lamu, the spread of Islam is linked to the Hadramy Alawi (Hadramaut) networks originating from Yemeni Sufism, along with the accompanying academics. While in Wajir the spread of Islam is associated with missionary excursions by Somali merchants and Sufi brotherhood Hadrami and other Arab traders through Ethiopia into towns in northern Kenya the former North eastern province which includes [27, 28].

### Sample size and sampling procedure

Data collection involved administration of a semi structured questionnaire to female respondents aged 18–49 years in their households (Additional file 1). We calculated samples size using formulae shown below adopted from UNICEF’s Multiple Indicator Cluster Surveys (MICS) [29].

$$n = \frac{{4 \times r \times (1 - r) \times deff}}{{{{(RME \times r)}^2} \times pb \times AveSize \times RR}}$$

Where n is the required sample size (number of households); 4 is a factor to achieve the 95% level of confidence; r is the predicted or estimated value of the indicator in target population; deff is the design effect; RR is the response rate; pb is the proportion of the target subpopulation in total population (upon which the indicator, r, is based); AveSize is the average household size (that is, average number of persons per household); RME is the relative margin of error to be tolerated at the 95% level of confidence; Currently, the target for the RME is generally is 12%. The total sample size for all domains was 350 in each county.

### Sampling strategy

The study used multi-stage sampling to select the respondents. In the first stage, each sub-county was stratified into constituent wards, sub-locations and further into villages. The village comprised the primary sampling units (PSU). From each village, we obtained a list of all households from the chief’s office. In the second stage, households were selected from the household listing in each selected PSU using systematic sampling with a random start; the random start was determined by randomly selecting a number. The inclusion criteria were women and girls aged 15–49 within the selected households. However, only one woman of reproductive age were interviewed per household. In households where more than one woman of reproductive age 15–49 resides, a Kish grid^3^ was used to select one respondent from that household. We excluded those who did not provide consent, and women aged < 18 (unless they were emancipated minors) or > 49 years old. Although minors below 18 were part of the inclusion criteria there were no minors interviewed. Although there was no open refusal of consent, the household did not declare any respondent aged 15–17 emancipated. This could be because family planning is already a highly sensitive issue in certain communities, and they are aware that child marriage which is one of the categories for emancipated minors in our case, is illegal.

### Data management and statistical analysis

Data were double entered and cleaned in epi infoTM 7 before statistical analyses. All analyses were conducted in STATA software version 15 (StataCorp. 2017). Descriptive statistics were used to summarise data whilst tables and figures were used for data presentation. Binary and multiple logistic regression analysis was used to identify the determinants of family planning use. Odds ratio with their corresponding 95% confidence intervals were used to determine magnitude and directions of the statistical associations in both bivariate and multivariable analyses. *p*-value was considered statistically significant at 0.05.

### Outcome variable

The study had one outcome variable which is current use of modern contraceptive method. They were coded as 1 “current use” and 0 otherwise. Current use was defined as women who were currently using any modern contraceptive method.

### Explanatory variables

The explanatory variables were chosen based on expert opinion or prior knowledge. They included; the age of the participant, level of education, marital status, age at marriage, employment status, discussion with a spouse, ever given birth, family planning awareness (able to list modern FP methods), number of living children, acceptability in culture, FP allowed in Islam and willingness to obtain information on FP.

### Data analysis

A descriptive analysis was carried out to establish the level of current use, method preference and reasons for non-use after which frequency and 95% confidence intervals were reported. The binary logistic regression analysis was used to determine the determinants associated with family planning use. Crude Odds ratios (COR) and Adjusted Odds Ratio (AOR) with their corresponding 95% confidence intervals were used to determine the magnitude and directions of the statistical associations in both bivariate and multivariable analyses. A *p*-value of < 0.05 was considered statistically significant.

### Ethical considerations

#### Ethical approval

for the study was obtained from the Research Ethics Committee of the Aga Khan University, Nairobi (2016/REC-56 (v3). We also obtained a research permit from the National Commission for Science, Technology and Innovation (NACOSTI/P/18/14,340/20,946) to facilitate the conduct of research activities in the community. All participants provided informed consent after being informed about the objective of the study, those who could not write used thumb prints. Regarding minors below the age of 18 years only married adolescents who are considered as emancipated, were considered in the inclusion criteria. .

## Results

### Socio-demographic and economic characteristics of the study participants

Of the 663 women of reproductive age consented to participate in the study,342 (51.5%) and 321 (48.5%) came from Lamu and Wajir County respectively. The median age of study participants was 30 years (IQR, 24–35) in Lamu and 28 (IQR, 25–34) in Wajir. The majority of the study participants (Lamu 51.6% vs. Wajir 48.4%) in the two counties were aged between 25 and 34 years of age. There was a significant difference by education level with the majority (72.2%) of the participant in Lamu County having secondary education and above while in Wajir County majority (54.2%) had no formal education. In Lamu 127 (44.2%) were married at the age of 15–19 years while in Wajir majority were married at the age of 20–24 years. There was significant difference in the distribution of study participants by age group (Table 1).

**Table 1:** Socio-demographic and economic characteristics of study participants (*N* = 663)

	County		
	**Lamu n=(342)**	**Wajir n= (321)**	P-value
Characteristic			
M*edian (IQR), years*	30 (24–35)	28 (25–34)	0.427
**Age categories (years)**:	n (%)	n (%)	
18–24	97 (28.6)	79 (24.6)	0.126
25–34	154 (45.0)	168 (52.3)	
35–44	80 (23.3)	70 (21.8)	
45+	11 (3.2)	4 (1.2)	
**Highest level of education**:			
None/Madrassa	25(7.31)	171 (53.3)	**< 0.0001**
Primary	70 (20.5)	71 (22.1)	
Secondary and above	247 (72.2)	79 (24.6)	
**Marital status**:			
Married	287 (83.9)	265 (82.6)	0.638
Not Married	55 (16.1)	56 (17.4)	
**Age at marriage –years** (***n*** = **552)**:			
<15	17 (5.9)	0 (0.0)	**< 0.0001**
15–19	127 (44.2)	84 (31.7)	
20–24	109 (37.9)	129 (48.6)	
25–34	33 (11.5)	52 (19.6)	
35+	1 (0.3)	0 (0.0)	
**Age at first married (M** ***ean ± SD, year)***	19.7 (3.7)	21.4 (3.3)	**< 0.0001**
**Currently employed**:			
Yes	58 (16.9)	52 (16.2)	0.793
No	284 (83.1)	269 (83.8)	

^**values in bold are statistically significant at p−value<0.05^

### FP use by methods

**Fig. 1:**
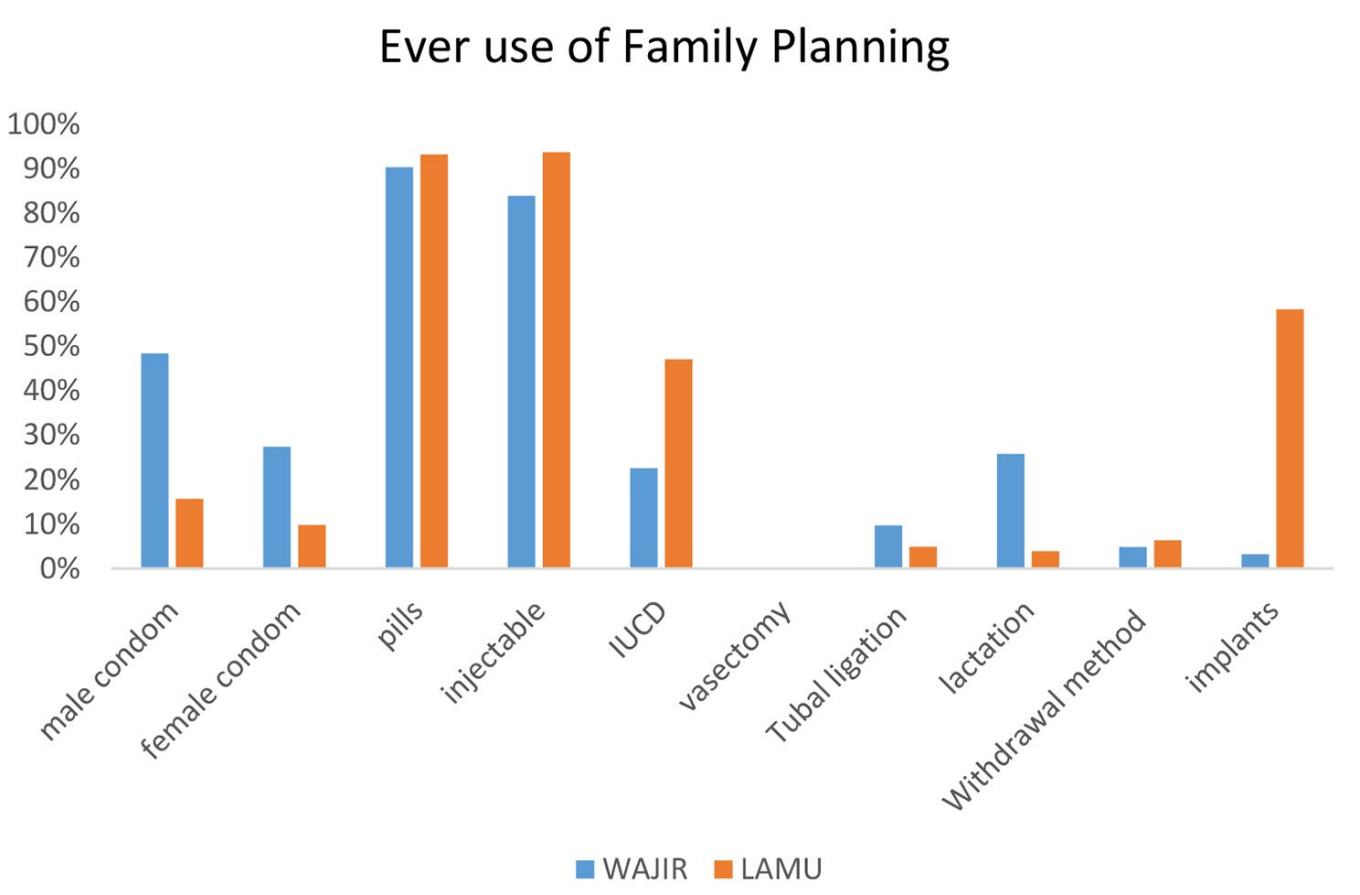
Ever Use of FP by county of residence (*N* = 266)

Figure 1 shows the type of family planning methods used by the participants’ county of residence. The most prevalent methods of family planning are pills and injectable. Among those who reported ever used family planning, 90% in Wajir and 93% in Lamu used contraceptive pills, while injectable use was at 94% in Lamu and 84% in Wajir. Further, implants were mostly used in Lamu (58%) as compared to Wajir at 3%. Both male and female condoms were used more in Lamu compared to Wajir. The utilization of Intrauterine Contraceptive Device (IUCD) was higher in Lamu at 47% compared to Wajir at 23%.

### Percentage of women currently using family planning

Table 2 shows the current use of family planning in the two study sites. The percentage of women currently using family planning was 18.6% (*n* = 123). In Lamu, the percentage was 32.8% while in Wajir, it was 3.4%. In Lamu, current use of FP was highest among women between 25 and 34 years with a percentage of 42.9% (35.2–50.8), while in Wajir, the highest FP use was among women aged 22–34 years at 5.4% (2.4–82.1). In Lamu, current FP use was highest among women who had attained secondary education at 34.3% (28.9–40.2), while in Wajir it was 9.8% (4.9–18.4). The current use of FP was highest among married women in both counties; 35.2%(29.9–40.9) in Lamu and 3.8% (2.0-6.9) in Wajir. In Lamu and Wajir, women who got married between the ages of 25 and 34 had the highest prevalence of current use of FP at 48.5% (31.9–65.4) and 5.8% (1.8–16.6), respectively. In both counties, more employed women were currently using FP compared to unemployed women.

**Table 2:** Percentage of women currently using Contraceptive in Lamu and Wajir

		County			
		Lamu (*n* = 342)		Wajir (*n* = 321)	P-value
Current FP (Overall % prev)		32.8		3.4	
**Variable**					
**Age categories (years)**:		n (%)		n (%)	
**18–24**	*n* = 342	24.7(17.1–34.4)	*n* = 321	1.3(0.2–8.6)	
**25–34**		42.9(35.2–50.8)		5.4(2.8–10.0)	**0.02**
**35–44**		24.2(16.4–34.1)		0	
**45+**		0		25.0(2.4–82.1)	
**Highest level of education**:					
**None/Madrassa**	*n* = 342	8.3(1.1–43.7)	*n* = 321	1.2(0.3–4.6)	
**Primary**		30.5(20.0-43.5)		1.5(0.2–9.9)	**< 0.0001**
**Secondary and above**		34.3(28.9–40.2)		9.8(4.9–18.4)	
**Marital status**:					
**Married**	*n* = 342	35.2(29.9–40.9)	*n* = 321	3.8(2.0-6.9)	**0.021**
**Not Married**		20.0(11.4–32.8)		1.8(0.2–11.9)	
**Age at marriage –years** :					
<25	*n* = 342	33.6(28.0-39.7)	*n* = 321	3.3(1.6–6.8)	
**25–34**		48.5(31.9–65.4)		5.8(1.8–16.6)	0.634
**> 35**		19.6(11.1–32.3)		1.8(0.2–11.9)	
**Currently employed**:					
**Yes**	*n* = 342	53.4(40.5–65.9)	*n* = 321	5.8(1.8,16.6)	
**No**		28.5(23.5–34.1)		3.0(1.5,5.9)	< **0.0001**
**Ever given birth**:					
**Yes**	*n* = 342	37.9(32.4–43.8)	*n* = 321	3.8(2.0-7.2)	
**No**		8.3(3.5–18.7)		2.4(0.6–9.1)	< **0.0001**
**Number of living children** :					
**0**	*n* = 235	28.6(6.3–70.4)	*n* = 215	16.7(3.9–49.6)	
**1**		34.6(25.0-45.6)		3.6(0.9–13.7)	0.007
**2–4**		48.3(39.4–57.4)		5.4(2.2–12.4)	
**5 or more**		24.1(11.8–43.1)		3.6(0.9–13.7)	
**Currently pregnant**:					
**Yes**	*n* = 342	10.4(5.0-20.5)	*n* = 321	3.5(0.9–13.2)	< **0.0001**
**No**		38.2(32.6–44.1)		3.4(1.8–6.4)	
**The current pregnancy is first pregnancy** :					
**Yes**	*n* = 67	0	*n* = 57	0	0.029
**No**		14.6(7.0–28.0)		5.7(1.4–21.0)	
**Ever discussed with partner on FP use**:					
**Yes**		46.2(39.3–53.2)	*n* = 321	6.4(2.7–14.6)	< **0.0001**
**No**	*n* = 342	14.5(10.0-21.2)		2.5(1.1–5.4)	
**Aware on FP methods**:					
**Yes**	*n* = 342	33.6(28.7–39.0)	*n* = 321	4.8(2.4–9.3)	< **0.0001**
**No**		19.1(7.1–41.9)		2.0(0.6–5.9)	
**FP acceptable in culture**					
**Yes**	*n* = 341	47.7(40.7–54.8)	*n* = 257	5.8(2.8–11.7)	
**No**		13.5(8.9–20.1)		2.2(0.7–6.7)	0.001
**FP is allowed in Islam**					
**Yes**	*n* = 324	37.0(30.0-44.6)	*n* = 171	13.4(7.1–24.0)	
**No**		29.6(23.0-37.2)		1.6(0.4–6.3)	0.029
**Willingness to obtain Information**					
**Yes**	*n* = 342	71.2(62.0-78.9)	*n* = 321	6.2(3.2–11.5)	
**No**		14.3(10.3–19.4)		1.1(0.3–4.5)	< **0.0001**

^**values in bold are statistically significant at p−value <0.05^

In Lamu, the percentage of employed women currently using FP was 53.5% (40.5–66.0), while in Wajir, it was 5.8% (1.8–16.6). Women who had ever given birth in Lamu and Wajir were the highest group currently using FP 37.9% (32.4–43.8) and 3.8% (2.0-7.2), respectively. In both counties, women who discussed FP use with their partners were current users of FP. In Lamu, the percentage of women currently using FP and discussed this with their partners was 46.2% (39.3–53.2), while in Wajir, it was 6.4% (2.7–14.6). In both counties, women who were aware of FP methods were currently using FP. The percentage of women who were aware and were currently using FP was 33.6% (28.7–39.0) in Lamu and 4.8% (2.4–9.3) in Wajir. We found that, the highest prevalence of current use of FP was among women who stated that FP use was acceptable in their culture; it was 47.7%(40.7–54.8) in Lamu while in Wajir, it was 5.8% (2.8–11.7). In Lamu, the current use of FP was highest among women who stated that the use of FP is allowed in Islam is 37.0%(30.0-44.6). Similarly, in Wajir, current use was highest among women who stated that FP was allowed in Islam, 13.4% (7.1–24.0). The highest prevalence in Lamu and Wajir was among women who were willing to obtain information regarding contraceptives 71.2% (62.0-78.9) and 6.2% (3.2–11.5) in Lamu and Wajir respectively.

### Reasons for family planning non-use

**Fig. 2:**
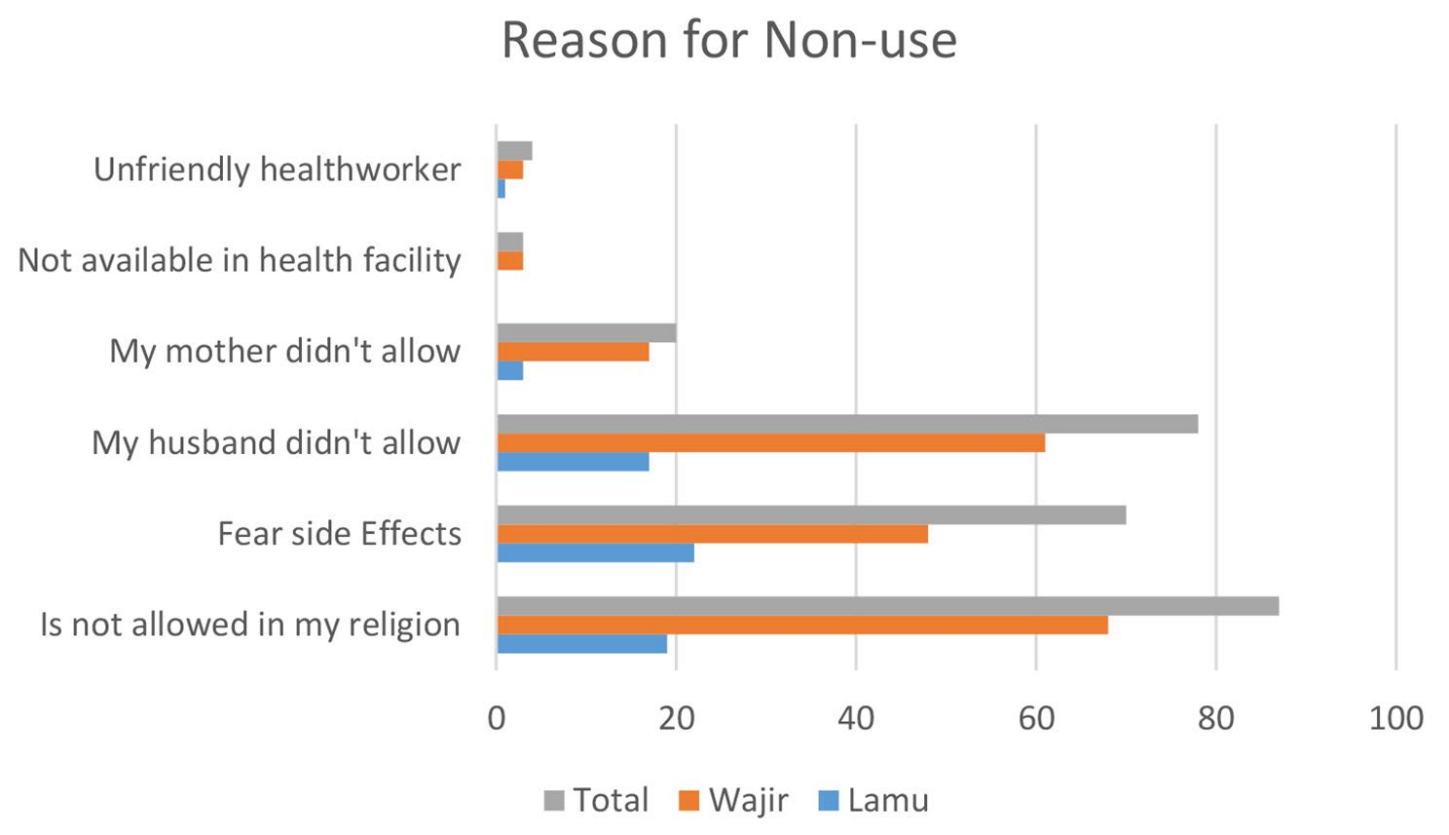
Reasons for Non-use

A total of 392 (59.13%) study participants reported they have never used any family planning methods. The common reasons for not using family planning methods were: belief that Islam does not allow (70.6%), the husband refused to use (78%%), fear of side effects (70%) and mother-in-law refused 5.73%). It is important to highlight that Wajir has the highest rate of nonuse for all factors. Belief that Islam prohibits the use of FP is higher in Wajir than in Lamu. The percentage in Lamu is 19%, while the percentage in Wajir is 68% in comparison. The percentage for fear of side effects is 48% in Wajir whereas in Lamu it was 22%. Refusal by husband for Wajir was at 61% compared to Lamu at 17%. Figure 2 shows the reasons for never using family planning methods.

### Determinants of current contraceptive use

Table 3 shows the results of a logistic regression analysis of determinants of current FP use. In Lamu the odds of the current use of FP is significantly higher for those in current employment compared to the unemployed (AOR = 3.06 95% CI = 1.30–7.19). Those who have ever discussed FP with their partners are more likely to be current users of family planning (AOR = 2.63 95% CI = 1.25–5.53). Women who believed that family planning is acceptable in their culture are more likely to be current users of family planning compared to those who don’t (AOR = 5.00 95% CI = 2.38–10.49). The odds of the current use of FP is significantly higher for those who are willing to obtain information compared to those not willing to obtain information (AOR = 20.04 95% CI = 9.77–41.10). In Wajir, factors identified as determinants of the current use of family planning were education level, FP allowed in Islam and willingness to obtain information. A woman with secondary level of education and above is 7.39 times more likely to be a current family planning user compared to women with no education (AOR = 7.39 95% CI = 1.42–38.60). Women who believed that family planning is allowed in Islam are more likely to be current users of family planning compared to those who don’t (AOR = 9.47 95% CI = 1.98–45.21). The odds of the current use of FP is significantly higher for those who are willing to obtain information compared to those not willing to obtain information (AOR = 2.88 95% CI = 0.52 = 15.94).

**Table 3:** Determinants of current use of contraception (*n* = 663)

	Lamu		Wajir	
	Outcome		Outcome	
Variable	COR(95% CI)	AOR(95% CI)	COR(95% CI)	AOR(95% CI)
Age category **(years)**:				
**< 25**	**REF**	REF	REF	REF
25–34	2.28(1.30,4.00)*	1.84(0.82,4.11)	4.42(0.55,35.47)	
35+	0.97(0.50,1.89)	0.61(0.24–1.60)	1.07(0.07,17.40)	
Level **of education**:				
**None/Madrassa**	**REF**	REF	REF	REF
Primary	4.83(0.58–40.27)		1.26(11.25–14.14)	0.69(0.06–8.23)
Secondary and above	5.75(0.73–45.20)		9.14(1.89–44.06)*	7.39(1.42–38.60)*
Marital status:				
**Not married**	**REF**	REF	REF	REF
Married	2.17(1.07,4.39)*		2.16(0.27,17.20)	
Age at **marriage**				
**< 25**	**REF**	REF	REF	REF
25–34	1.86(0.90,3.86)	1.09(0.34–3.56)	1.80(0.45,7.22)	
35+	0.48(0.24,0.98)*	0.23(0.08–0.68)*	0.54(0.64,4.44)	
Currently **employed**:				
**No**	**REF**	REF	REF	REF
Yes	2.88(1.62,5.12)*	3.06(1.30,7.19)*	2.00(0.51,7.79)	
Ever discussed **with partner on FP use**:				
**No**	**REF**	REF	REF	REF
Yes	5.07(2.95,8.70)*	2.63(1.25–5.53)*	2.71(0.80,9.12)	
Ever given **birth**:				
**No**	**REF**	REF	REF	REF
Yes	6.73(2.61,17.33)*	2.56(0.78–8.48)	1.62(0.34,7.65)	
Number of **living children (*N*** = **431)**:				
**0**	**REF**	REF	REF	REF
1	1.32(0.24–7.25)		0.19(0.02–1.50)	
2–4	2.34(0.44–12.52)		0.28(0.05–1.66)	
5 or more	0.80(12.54–5.05)		0.19(0.02–1.50)	
FP awareness				
**No**	**REF**	REF	REF	
Yes	2.15(0.71,6.56)		2.5(0.65,9.60)	
FP **acceptable in my culture**:				
**No**	**REF**	REF	REF	REF
Yes	5.83(3.37–10.10)*	5.00(2.38–10.49)*	2.72(0.69–10.77)	
FP is **allowed in Islam**:				
**No**	**REF**	REF	REF	REF
Yes	1.40(0.88–2.22)		9.47(1.98–45.21)*	8.36(1.55–45.01)
Willingness				
**No**	**REF**	REF	REF	REF
Yes	14.81(8.53–25.72)*	20.04(9.77–41.10)*	5.76(1.22–27.09)	2.88(0.52–15.94)

^Values in * show significance at p−value <0.05^

## Discussion

This study aimed to explore the pattern and determinants of contraceptive use among women in Wajir and Lamu counties in Kenya. The percentage of the current use of FP in Lamu was 32.8% while in Wajir it was 3.4% percent. This finding is similar to the 2014 and 2023 KDHS results [6, 7].

The findings illustrate that current contraceptive use in Lamu was significantly influenced by; employment, discussion of family planning with a partner and ever given birth. In Wajir, the significant factors were; education, discussion with partner; FP allowed in Islam and willingness to obtain information on FP.

The study results also revealed that in both counties injectable were the most common method of contraception 94% and 84% in Wajir and Lamu respectively. This was then followed by pills at 93% and 90% in Lamu and Wajir respectively. These finding are similar to the KDHS 2014 report and a study conducted in Ghana that revealed that injectable are the most common family planning method among women [7, 30]. The preference for injectable could be attributed to lack of partner support and the fact that injectable can be used covertly. In one qualitative study conducted in Ghana, respondents reported that uptake of family planning was perceived to be associated with promiscuity and this could explain why most women in this study preferred the use of injectable which gives women the autonomy for covert use [31].

The descriptive analysis show that 59.13% of the respondent indicated that they have never used any FP method. The highest proportion of those who indicated to have never used any contraceptive method were from Wajir county 79.13%. The leading reason for non-use were; the belief that religion does not allow FP use (70.6%) and husband’s disapproval (22.14%). The notion that Islam does not permit the use of FP could be one of the reasons for low contraceptive use among women in Wajir with 68% of the respondent indicating Islam is against the use of FP. The misconception that Islam does not allow FP use has been documented in other studies [32–35].

The influence of men in women’s ability to decide whether to use contraception is evident from our results with overall 78% indicating husband’s disapproval as reason for non-use. Opposition of FP use by spouse is higher in Wajir compared to Lamu at 61% and 17 respectively. This depicts the dominance of men in decision making in patriarchal African society. This illustrates patriarchal structures and values that hinder the autonomy of women in relation to their reproductive health particularly in Wajir. These findings are comparable to an earlier qualitative study conducted in the two counties which although men indicate family planning is a women’s affair they still want to be consulted and be the final decision makers on whether or not family planning [21, 33].

Our results show that, married women in Lamu are nearly three times more likely to be current users of family planning compared to non-married women. This is consistent with a study conducted in Kenya and in Uganda [19, 36]. For instance the 2014 KDHS report states that 53% of currently married women are using contraceptives [7]. Contraceptive use is higher among married women because they are more likely to be sexually active therefore at risk of getting unplanned pregnancies.

The odds of current use of family planning is higher among women who are currently employed in Lamu compared to those who are unemployed. This is consistent with other studies conducted that focused on the determinants and prevalence of contraception use among employed and unemployed women [8, 37]. Employed women are more likely to use contraception because they have more negotiation power on contraceptive use.

Our findings demonstrate that in both study sites, women who discussed FP with their partners are more likely to be current users of contraceptive. Our results are in agreement with several studies that show direct correlation between FP use and discussion with partner [30, 38, 39]. A study conducted in southeast Ethiopia revealed that opposition to modern family planning by spouses determined whether they used contraception [40].

The findings revealed that in Wajir, women who had above the secondary level of education were more likely to be current users of FP compared to those who had not. This is consistent with several studies [8, 14]. These results could be explained by the fact that educated women are more informed about their reproductive health, understand the benefits and the side effects that come with the use of family planning. Educated women are also empowered, giving them more authority to make decisions regarding their reproductive health. Wajir county has one of the lowest educational attainment levels in Kenya with as many as 76% of its residents having low education levels and only 4% have completed secondary level education or higher [25]. The low education level particularly for women and girls could be one of contributing factors to low uptake of family planning in Wajir county.

The results revealed that in Lamu, culture was a key determinant of FP utilization while in Wajir permissibility by Islam was a key determinant of FP uptake. Though the position of Islam regarding family planning is clear, studies have shown that there is misinterpretation informed by local cultures. A qualitative study conducted in Wajir and Lamu counties of Kenya revealed the labelling of cultural values as religious teachings mainly due to limited understanding of Islamic doctrines [21, 33].Many studies and analytical reviews show that Islam allows the use of family planning within the confines of marriage. Various scholarly sources provide evidence indicating that Islam does not prohibit the utilization of contraceptives. The first source of Islamic sharia, the Quran, prescribes that mothers should breastfeed for a duration of two full years [41, 42]. Similarly, the Sunnah which serves as a record of the traditions of the Prophet Muhamad Peace Be Upon Him (PBUH), provides evidence that coitus interruptus, also known as withdrawal or al Azl, was used during the era of the prophet (PBUH). Analogical reasoning (qiyas), which is considered the third source of Islamic sharia, has been employed by numerous Muslim scholars to establish the legitimacy of reversible contraceptive methods. This is due to the fact that both traditional methods such coitus interruptus and modern contraceptive techniques share the common objective of preventing conception [43–45].

Although there is a consensus among Muslim scholars on permissibility of family planning in Islam, myths and misinterpretations regarding Islam and family planning persist. The misconception of religion, specifically within the Somali community, has been linked to a limited comprehension of Islamic teachings. Certain practises, such as Female Genital Mutilation, are erroneously regarded as religious practises while lacking any foundation in Islam. Instead, they are rooted in cultural traditions. This observation highlights the nuanced relationship between religion and culture. Where there is a controversy between culture and religion, in many instances cultural practices are branded as religious [21, 46]. The concept that Islam opposes family planning may serve as a significant obstacle to contraceptive uptake within Wajir County, which is primarily inhabited by the Somali community. The phenomenon of low contraceptive utilisation among the Somali population has been extensively documented through several studies conducted both within Somalia and among Somalis residing in diaspora settings. The contraceptives prevalence rates in Somalia, for instance, stands at 1% [47]. Research undertaken in Norway, Finland, and the United States among the Somali population has revealed a notable low utilisation of family planning methods, as well as a strong inclination towards having larger families. This preference for larger families is often influenced by societal norms that assess the social status and standing of women based on the number of children they bear [35, 48, 49].

The higher contraceptive prevalence rate in Lamu may be attributed to the comparatively greater educational achievement in comparison to Wajir. Moreover, it is worth noting that Lamu County had been a prominent hub for Islamic education within Kenya and the broader eastern African region. This can be attributed to the notable Riyadha mosque and the significant influence exerted by the Hadramy Alawi (Hadramaut) networks originating from Yemeni Sufism, along with the accompanying academics. This advance knowledge of Islam could have contributed correct interpretation of Family planning from an Islamic perspective [28].

While our study has identified some significant determinants of family planning, it is important to acknowledge that there are additional crucial determinants that were not specifically addressed in our research. Previous studies have demonstrated the significance of factors such as access to family planning commodities and the attitudes of healthcare providers as key determinants. Study conducted in Ethiopia revealed that 43% of study participants indicated that they could not get the method they prefer in the nearby health facility [50]. Health care practitioners may be impacted by their personal biases, which may deny clients access to their preferred methods of contraceptive choice. A study conducted in Ghana revealed that healthcare practitioners prioritised highlighting the potential adverse effects of the contraceptive pill, rather than presenting it as an initial option for contraception. Additionally a number of providers apparently believed that injectable contraceptives cause permanent infertility [51].

Whereas our study has unearthed some key factors that determine family planning uptake among Muslim women in the two counties, it is worth noting that this study had its own limitations. Like many other cross sectionals studies, we were not able to measure cause and effect relationship. Although we included adolescent girls below the age of 18 in the inclusion criteria, there were no adolescent respondents below the age of 18. The lack of adolescent respondents could be attributed to the sensitivity around contraceptive use among these age group in an Islamic setting. This is an area that we recommend for further research. However, despite these shortcomings the paper provides an interesting contribution in understanding the pattern and determinants of family planning uptake among Muslim communities in an area that has not been fully explored in the Kenyan context.

### Conclusion and recommendation

We found moderately high use of contraceptive use among women of reproductive age in Lamu county and very low current contraceptive use among women in Wajir. Education has shown to be a key determinant in decision-making on contraceptive use particularly in Wajir which has one of the lowest education attainment levels in the country, and thus it is important for the national and county government to invest in education, particularly for women and girls to enhance their ability to make informed decisions. Discussion with partner has come out as a key determinant for FP use in both counties. These findings reflect the need for family planning programs to address issues of male involvement in reproductive health programmes given the patriarchal nature of the two communities. Further, our findings highlight the need to develop culturally appropriate messages and involvement of religious leaders to demystify the myths and misconception around family planning and Islam especially in Wajir. It is worth exploring inter-county learning and exchange programme for Wajir County to learn from Lamu given the homogeneity in religious beliefs.

### Electronic supplementary material

Below is the link to the electronic supplementary material.

Supplementary Material 1

## Data Availability

The datasets used to analyze the current study are available from the corresponding author on reasonable request.

## Footnotes

1 Fatwa- authoritative legal opinion or learned interpretation by a qualified jurist regarding issues pertaining to Islamic Shariah.

2 **Sources of Islamic law (Shariah)**: The primary sources of Shariah are the Quran (Islam’s holy book) and the Sunnah, the sayings and deeds of the Prophet Mohammad and his Companions. Also based on the Quran and Sunnah but subordinate to them are two other sources for Shariah: the consensus of Islamic jurists (ijmae) and analogy (qiyas).

3 The Kish grid is a method for selecting members within a household to be interviewed. It uses a pre-assigned table of random numbers to find the person to be interviewed.

## Abbreviations

CPR: Contraceptive prevalence rate
DHS: Demographic and health survey
FP: Family Pplanning
ICD: Intrauterine Contraceptive Device
KDHS: Kenya demographic and health survey
MICS: Multiple Indicator Cluster Survey
NACOSTI: National Commission for Science, Technology and Innovation
TRF: Total fertility Rate
UNFPA: United Nations Population Fund
