# Supplementary material for: Pattern and determinants of contraceptive use among the muslim women in Wajir and Lamu counties in Kenya: a cross-sectional study
Source: BMC Womens Health. 2024 Jan 18;24:53. doi: 10.1186/s12905-024-02892-9 (PMC10795387; doi:10.1186/s12905-024-02892-9)
Supplement: Supplementary file 1 — Supplementary Material 1 [file 12905_2024_2892_MOESM1_ESM.doc]

1. **Survey of women in Reproductive age**

**INTERVIEWER (DO NOT READ):**

Ensure to start with the following

**1.** Greetings and introduction

**2.** Informed consent – **NB**: Provide copy of consent, contact information.

| **Serial No** | | | |
| --- | --- | --- | --- |
| **Area/Region** |  | **Date of Interview** | **__ __ / __ __ / 2018**  **(DD/MM)** |
| **Name of community**  **Or Area** |  | **Interviewer Name** |  |
| **Start Time** | **___ ___ : ___ ___** | **End Time** | **___ ___ : ___ ___** |
| **Interviewer observations (please enter relevant observations about the interview)** | | | |

| SECTION 1 – GENERAL BACKGROUND INFORMATION - Social demographic data | | | | | | | | |
| --- | --- | --- | --- | --- | --- | --- | --- | --- |
| **First I have some general questions about you, your family and circumstances.** | | | | | | | | |
| **#** | | **QUESTIONS** | | **RESPONSE CATEGORIES** | | | **RESPONSE** | |
| 101a | | **In what month and year were you born?**  **The age in the household roster is [AGE].** | |  | | | Month  _][__]  DK in month not known.. code as 98  Year  _][__]_][__]  Enter DK if year is not known and enter or circle code 9998 | |
| 101b | | **How old were you at your last birthday? Or How old are you now?**  **Compare and correct age above if inconsistent**  Age | | _______________________________________ | | | [__][__] years | |
| 102 | | Have you ever been married | | Yes  No | | |  | |
| 103 | | What is your marital status? | | Single  Married  Separated but living with someone  Divorced  Widowed  Other __________________ | | | 1  2  3  4  5  6 | |
| 104 | | How old were you when you got married | |  | | | [__][__] years  DK | |
| 105 | | Have you ever attended school or pre school | | Yes  No | | |  | |
| 106a | | What is the highest level of education you reached? | | University  College (diploma certificate)  Secondary and above  Primary complete  Primary Incomplete  Madarasa (Islamic education)  No education | | | 1  2  3  4  5  6  7 | |
| 106b | What is the highest grade you completed at that level | | Grade …… | | |  | | |
| 107 a | Check for 106 or 106b if sec and above **SKIP to 108**  If primary and below continue to next question | |  | | |  | | |
| 107b | Now I would like you to read this sentence to me  Show the sentence on the card to respondent. If the respondent cannot read whole sentence probe:  Cannot read part of sentence to me | | Cannot read at all  Able to read only parts of sentence  Able to read whole sentence  No sentence in required language-------( specify language  Blind/visually impaired | | | 1  2  3  4  5 | | |
| 108 | Now I would like to ask about all births you have had during your life.  Have you ever given birth? | | Yes  No **SKIP to 118** | | | 1  2 | | |
| 109 | What was the date of your first birth?  I mean the very first time you gave birth even if the child is no longer living | | Date of first birth | | | Month  _][__]  DK in month not known. code as 98  Year  _][__]_][__]  Enter DK if year is not known and enter or circle code 9998 | | |
| 110 | How many years ago did you have your first birth | | Completed years since the first birth | | | [__][__] years | | |
| 111 | Do you have any sons or daughters to whom you have given birth who are living with you? | | Yes  No | | |  | | |
| 112 | How many sons live with you?  How many daughters live with you | | Sons  Daughters | | | Sons [__]  Daughters [__] | | |
| 113 | Do you have any sons or daughters to whom you have given birth to who are alive but not living with you? | | Yes  No-Skip to 115 | | |  | | |
| 114 | How many sons are alive but not living with you?  How many daughters are alive but not living with you? | | Sons elsewhere  Daughters elsewhere | | | Sons [__]  Daughters [__] | | |
| 115 | Have you given birth to a boy or a girl who was born but later died | | Yes  No-skip to 117 | | |  | | |
| 116 | How many boys have died?  How many girls have died? | | Boys died  Girls died | | | Boys died [__]  Girls died [__] | | |
| 117 | Of these births you have had, when did you deliver the last one? ( even if he or she died) | | Date of last birth  Month  Year | | | Month [__] [__]  DK 98  Year  [__] [__][__]_][__] | | |
| 118 | Are you currently employed? | | Yes  **No SKIP to 120** | | |  | | |
| 119 | If Yes what is your occupation | |  | | |  | | |
| 120 | Do you have a consistent source of income? | | Yes  No | | |  | | |
| 121 | If yes what is the source of your income | | Record responses | | |  | | |
| SECTION 2 – Family planning Services Knowledge, Attitude, Perceptions, Preferences | | | | | | | | |
| I am now going to ask you about Family Planning/Child spacing | | | | | | | | |
|  | | **QUESTIONS** | | **RESPONSE CATEGORIES** | | | **RESPONSE** | |
| 201 | | Are you currently Pregnant? | | Yes  No-Skip to 204  Don’t Know  Refused to answer | | | 1  2  3  4 | |
| 202 | | Was the pregnancy planned | | Yes  No  Refused to answer | | | 1  2  3 | |
| 203 | | Is this your first pregnancy? | | Yes  No  Refused to answer | | | 1  2  3 | |
| 204 | | Have you ever heard of Family planning/Child spacing | | Yes  No ***( proceed to 219)*** | | | 1  2 | |
| 205 | | Where/whom did you hear it from? | | Sister/ brother  Parent  Religious leader  Television (TV)  Radio  At school  Friend  Health facility/worker  Other______________________________ | | | 1  2  3  4  5  6  7  8  9 | |
| 206 | | What is family planning/child spacing? | | A away of spacing pregnancies  A way of stopping women from having children  Others (specify)…………………… | | | 1  2  3 | |
| 207 | | Please tell me all the methods of family planning you know or heard about.  ***DO NOT READ OUT LIST.***  ***MULTIPLE ANSWERS POSSIBLE.***  **PROBE ONLY WITH “ANY OTHER WAYS?”** | | Male Condom  Female Condom  Oral pills  Injectable  Intrauterine devices (IUCD)  Vasectomy  Tubal ligation  Lactation Amenorrhea (Breastfeeding)  Coitus Interruptus  Implants  Other (Specify) _____________________  Don’t know | | | 1  2  3  4  5  6  7  8  9  10  11 | |
| 208 | | How do married women in this community space their children/pregnancies | | _______________________________________________________________________________________________________________ | | |  | |
| 209 | | What do you think are the benefits of spacing children (Family planning) | | Spacing children helps the mother to regenerate her body and health  Children can have sufficient time to breastfeed and grow healthy  Small family size is easier to feed and educate  Others specify)…………………………………  No benefits | | | 1  2  3  4  5 | |
| 210 | | What do you think are the disadvantages of spacing once children (Family Planning) | | It has side effects like bleeding  It can make a woman infertile  It is against our religion  It is against our culture  Others (specify)………………… | | | 1  2  3  4  5 | |
| 211 | | What groups of people do you think should use Family planning methods  ***DO NOT READ OUT LIST.***  ***MULTIPLE ANSWERS POSSIBLE.***  **PROBE ONLY WITH “ANYONE ELSE?”** | | Young men  Young women  Older men  Older women  Commercial Sex workers  Married women  Married men  All people  Other (Specify) _____________________  Don’t know | | | 1  2  3  4  5  6  7  8  9  10 | |
| 212 | | Is the use of FP acceptable in your culture? | | Yes/no | | |  | |
| 213 | | If no, why? | | Indicate responses | | |  | |
| 214 | | According to your knowledge, is the use of FP allowed in Islam | | Yes  No | | |  | |
| 215 | | Are there any specific family planning method that are not acceptable in Islam | | Record responses | | |  | |
| 216 | | Is there any specific time limit for spacing/FP | | one year  two years  Five years  Depends on Family choice and circumstance | | |  | |
| 217 | | Have you ever talked to a religious scholar about the use of FP? | | Yes  No SKIP to 219 | | |  | |
| 218 | | If yes, what was their view? | |  | | |  | |
| 219 | | Which one is ideal time to have first child? | | 1. Between 18-21 2. Between 22-24 3. Between 25-27 4. Between 28-30 5. Over 30 | | | 1  2  3  4  5 | |
| 220 | | What is the ideal age space between children? | | 1. One year 2. One and half to two years 3. Three to five years 4. Five years or more | | | 1  2  3  4 | |
| SECTION 3 – Barriers/Challenges to Family Planning services | | | | | | | | |
| I am now going to ask you about utilization of Family planning services | | | | | | | | |
| **#** | | **QUESTIONS** | | **RESPONSE CATEGORIES** | | | | **RESPONSE** |
| 301 | | Can a person like you use family Planning services | | Yes  No | | | | 1  2 |
| 302 | | Have you ever used any Family planning method? | | Yes  NO ***( Skip to Q306)*** | | | | 1  2 |
| 303 | | Are you currently using any family planning method ( don’t ask if yes to question 201 | | Yes  No  Refused to answer | | | | 1  2  3 |
| 304 | | If you have discontinued what are the reasons | | a) wanted another child  b) Side effects  c) pressure from husband  c)Pressure from other family members  d) Other specify | | | | 1  2  3  4 |
| 305 | | Which Family planning method did you use? | | Male Condom  Female Condom  Oral pills  Injectable  Intrauterine devices (IUCD)  Vasectomy  Tubal ligation  Coitus Interruptus  Implants  LAM  Other (Specify) _____________________  Don’t know | | | | 1  2  3  4  5  6  7  8  10 |
| 306 | | Why didn’t you use it? | | Is not allowed in my religion  Fear side effects  My husband didn’t allow me  My Mother didn’t allow me  Was not available at nearby health facility  The health worker was unfriendly  Other (Specify)__________________ | | | | 1  2  3  4  5  6  7 |
| 307 | | Did you discuss it with your spouse or family members | | Yes (***Jump to Q309)***  No | | | | 1  2 |
| 308 | | If NO, Why? | | He will disapprove  He will beat me  He will divorce me  He wants to have more children  Others------------------------ | | | | 1  2  3  4 |
| 309 | | If YES, what was his reaction? | | Was supportive  Complaint to my family  Threw away my pills  No reaction | | | | 1  2  3  4 |
| 310 | | Do you personally know anyone who is using family planning services | | Yes  No ***(skip to Q312)*** | | | | 1  2 |
| 311 | | What is your relationship with this person/these persons?  **Multiple answers possible**  **Circle *all* mentioned** | | Close family member  Other relative  Close friend  Friend  Acquaintance  Neighbor  Other (specify)_____________________ | | | | 1  2  3  4  5  6  7 |
| 311 | | Do you think most of your close friends use family planning methods? | | Yes  No  DON’T KNOW | | | | 1  2  95 |
| 312 | | How do people in your community perceive people using child spacing?  **Probe with “ANY OTHER”** | | 1._________________________________________________________  2___________________________________________________.______  3_________________________________________________________  4_________________________________________________________ | | | | |
| 313 | | What is the main barrier to FP use in your community? | | Cost  Cultural conflict  Men do not allow their wives  Individual preference  Religion | 1  2  3  4  5 | | | |
| 314 | | Is the cost of FP services an obstacle | | Yes/NO |  | | | |

| **SECTION 4 – Family Planning Services Access & Recommendation** | | | |
| --- | --- | --- | --- |
| **Now I am going to ask you some questions about how easy or hard it is to get FP services around here** | | | |
| **#** | **QUESTIONS** | **RESPONSE CATEGORIES** | **RESPONSE** |
| 401 | Where is the nearest place to get mother and child services in this area? |  |  |
| 402 | Are there times when the family planning commodities run out | Yes  No  Don’t know | 1  2  3 |
| 403 | If you wanted to start using family planning services, where would you go?  **Do not read out. Select all that apply.** | Nearby government hospital/Health Centre  Private doctor/Health facility  Pharmacy  Other (specify) ______________________  Don’t know | 1  2  3  4  6 |
| 404 | According to you, what should be done to encourage women to seek family planning services | 1.__________________________________________________________________________  2.___________________________________________________________________________3.___________________________________________________________________________ |  |
| 405 | How would you like to hear/receive information on Family Planning- probe why the mode of choice. | TV  Radio  Posters  Written information  Personal interaction with someone  Knowledgeable  I don’t want to hear any information about Family planning  Don’t know | 1  2  3  4  5  6  7  8 |
| 406 | Why is that? Why would you like such mode of receiving information? |  |  |
| 407 | What sort of person would you most prefer to receive information from about Family Planning? | A Nurse  A Local Governemnt leader – chief  A religious leader  Someone I trust  Someone like myself but who knows a lot  I don’t care  I don’t want to hear anything about FP  Others_________________________ | 1  2  3  4  5  6  7  8 |

**Thank you for your time and cooperation.**
